# Epidemiology, clinical characteristics, flares and mortality of generalized pustular psoriasis: a nationwide register study in Finland

**DOI:** 10.1093/skinhd/vzaf076

**Published:** 2025-10-09

**Authors:** Mirkka Koivusalo, Xavier Teitsma, Juha Mehtälä, Aino Vesikansa, Susanne Clemen Capion, Laura Airaksinen, Maria Grönman, Rafael Pasternack, Pauliina Nuutinen, Laura Huilaja

**Affiliations:** MedEngine Oy, Helsinki, Finland; Boehringer Ingelheim B.V., Amsterdam, The Netherlands; MedEngine Oy, Helsinki, Finland; MedEngine Oy, Helsinki, Finland; Boehringer Ingelheim, Copenhagen, Denmark; Boehringer Ingelheim Ky, Helsinki, Finland; Boehringer Ingelheim Ky, Helsinki, Finland; Tampere University Hospital and Faculty of Medicine and Health Technology, Tampere University, Tampere, Finland; Skin and Allergy Hospital, Helsinki University Hospital, Helsinki, Finland; Research Unit of Clinical Medicine, University of Oulu and Medical Research Center, Oulu University Hospital, Oulu, Finland

## Abstract

**Background:**

Generalized pustular psoriasis (GPP) is a chronic and potentially life-threatening inflammatory disease characterized by recurrent, sudden flares often accompanied by systemic symptoms. With the introduction of new, targeted therapies for GPP there is an increased need to better understand patient characteristics to improve disease management.

**Objectives:**

To assess the epidemiology of GPP, and compare clinical characteristics, treatments and overall survival between GPP and population-based controls and control participants with psoriasis vulgaris. Furthermore, flare occurrence and risk factors for flares were also assessed.

**Methods:**

All Finnish patients with ≥1 GPP diagnosis (International Classification of Diseases, Tenth Revision: L40.1) in secondary healthcare between 1996 and 2021 and age- and sex-matched control groups were identified. Data on diagnoses, medications and deaths were collected from national registers. Incidence and prevalence were assessed using different case criteria. The primary analysis group included patients with ≥2 GPP diagnoses at a dermatology clinic in specialty care.

**Results:**

GPP period prevalence decreased from 9.9 to 4.7 and the incidence rate from 4.2 to 2.1 per 100 000 when using stricter case criteria. Psoriasis (73%) and hypertension (49%) were the most common comorbidities among patients with GPP, and topical corticosteroids were the most used medication (92%; *P* < 0.05 vs. control groups). Overall survival was lower in GPP compared with both control groups (*P* < 0.001). In total, 43% of the patients experienced a flare during follow-up, of which one-third (33%) had a flare at the time of first GPP diagnosis. Systemic corticosteroid use [hazard ratio (HR) 1.41; 95% confidence interval (CI) 1.10–1.81; *P* = 0.007] was associated with a higher risk of GPP flares and flares were associated with an increased risk of death (HR 1.7; 95% CI 1.08–2.60; *P* < 0.05).

**Conclusions:**

The epidemiology of GPP in Finland was found to be comparable to previously published estimates and associated with a high disease burden and shorter overall survival. Flares occurred frequently during follow-up and are associated with an increased risk of death. Standardized guidelines are crucial to improve the timely diagnosis and management of the disease.

## Teaser

Generalized pustular psoriasis (GPP) is a chronic and potentially life-threatening inflammatory disease characterized by recurrent, sudden flares and often accompanied by systemic symptoms. In this Finnish nationwide retrospective study, GPP prevalence and incidence rates decreased when using stricter criteria. Psoriasis and hypertension were the most common comorbidities, and topical corticosteroids was the most prescribed medication. One-third of patients had a flare at the time of first GPP diagnosis and flares were associated with an increased risk of death. Overall survival of GPP patients was lower compared with population-based and psoriasis vulgaris–matched controls. Standardized guidelines are crucial to improve the timely diagnosis and disease management.

What is already known about this topic?

- Estimates of the prevalence and incidence of generalized pustular psoriasis (GPP) vary due to rarity of the disease and differences in methodology used in patient identification.
- GPP flares are the main contributor to the disease burden, but real-world studies characterizing patients with flares are still scarce.
- The introduction of new, targeted therapies has increased the need for better understanding of patient characteristics to enhance personalized healthcare.

What does this study add?

- This is the first study to characterize the epidemiology, comorbidities, treatments, flares and mortality of GPP in Finland.
- The results show that disease burden and mortality rate are significantly higher in GPP compared with psoriasis vulgaris, and that flares are associated with a higher risk of death.
- Most flares occur at the time of first GPP diagnosis or shortly thereafter, emphasizing the importance of timely diagnosis and disease management.

Generalized pustular psoriasis (GPP) is a chronic, systemic, neutrophilic inflammatory skin disease characterized by widespread sterile pustules and systemic inflammation.^1–4^ While psoriasis vulgaris (PV), also known as plaque psoriasis, is the most common form of psoriasis, GPP is recognized as a distinct clinical entity with different pathophysiological mechanisms, including dysregulation of the interleukin (IL)-36 pathway. GPP can occur independently or in patients with a history of PV.^5^ The clinical course is complicated by intermittent episodes of flares, which are accompanied by systemic symptoms, and potentially life-threatening extracutaneous complications, including sepsis and multiorgan failure.^1–3,6–8^ Between 18% and 60% of patients with GPP experience flares, typically shortly after the first diagnosis,^8–11^ but the frequency, duration and severity of flares vary between patients and episodes.^12,13^ Previous studies have reported GPP prevalence estimates ranging from 0.18 to 18 per 100 000 individuals, depending on the criteria and methodology used for patient identification.^6,7,11,14–16^

GPP is associated with a significant disease burden and shorter survival, which is exacerbated by multiple comorbidities.^3,7,9,17,18^ The main driver of disease burden is recurrent flares, resulting in hospitalization in 50–90% of cases.^7–9,12,19^ Flares can be triggered by factors such as PV, obesity, alcohol use, electrolyte disorder and respiratory tract infections.^6–8,12,20^ Withdrawals and additions of certain medications, some of which are used to treat GPP, can trigger flares.^6–8,21,22^ Evidence suggests that GPP flares can directly contribute to mortality in hospitalized patients,^12^ and the overall mortality in GPP has been reported to be up to 50% higher than in PV.^11,23^

GPP treatment is largely based on controlling symptoms by using conventional antipsoriasis drugs.^2,6,8,9,24,25^ Despite receiving treatment with these immunomodulatory therapies, many patients experience ongoing chronic symptoms resulting in the need for more adequate treatment options.^6,25^ Biologics such as tumour necrosis factor-α and interleukin antagonists have been approved in Japan, Taiwan and Thailand for the treatment of GPP but not in other parts of the world due to lack of compelling evidence.^6,22,24,25^ In Europe and the USA, spesolimab, a selective humanized monoclonal antibody targeting the IL-36 receptor, was recently approved as the first targeted therapy for the continuous treatment of GPP.^13,26–28^

With the recent introduction of targeted therapies for GPP, there is an increased need to better understand patient characteristics and their clinical outcomes, and to form consensus criteria for the identification and diagnosis of GPP.^14,16^ However, due to the rarity and heterogeneous clinical course of GPP, comprehensive epidemiological and clinical data in the current literature are scarce. Population-based registry studies on patient characteristics have emerged in recent years,^9,14,16–18,23,29,30^ but, to the best of our knowledge, only few registry studies so far describe the real-world occurrence of GPP flares and its risk factors.^9–11^

This population-based, retrospective registry study aimed to assess the epidemiology of GPP in Finland, and to compare clinical characteristics, treatments and mortality between patients with GPP and matched control groups. Furthermore, we evaluated flare occurrence among patients with GPP, compared patients with and without flares, and assessed risk factors for flares.

## Patients and methods

### Study populations

**Figure 1:**
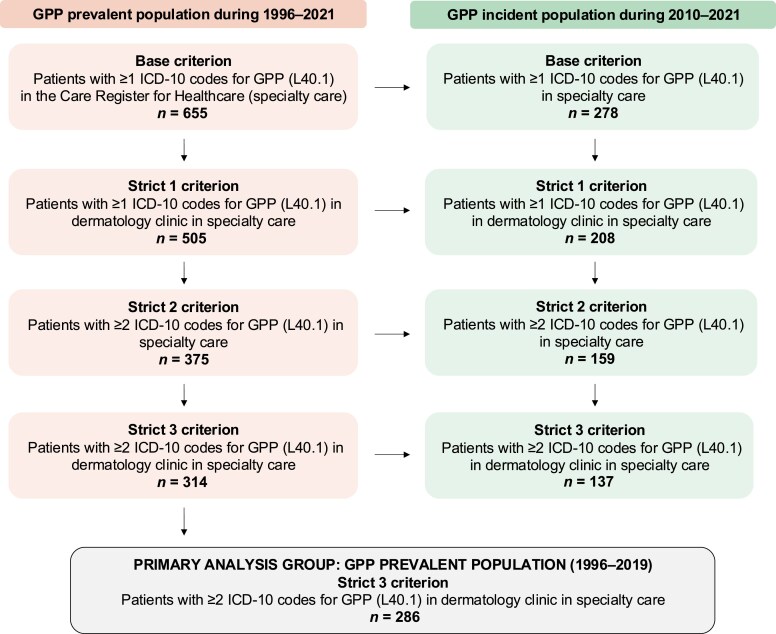
Flowchart of the inclusion and exclusion of the study population with generalized pustular psoriasis. ICD-10, International Classification of Diseases, Tenth Revision.

This was a nationwide noninterventional retrospective study that used data from Finnish national healthcare registers. The study cohort (base criterion) included all patients with ≥1 GPP diagnosis [International Classification of Diseases, Tenth Revision (ICD-10): L40.1] between 1996 and 2021 in specialty care (The Care Register for Health Care/The Finnish Institute for Health and Welfare). Three subgroups were formed with the following criteria: strict 1, ≥1 GPP diagnosis at a dermatology clinic in specialty care; strict 2, ≥2 GPP diagnoses in specialty care; and strict 3, ≥2 GPP diagnoses at a dermatology clinic in specialty care (Figure 1). The index date and the start of follow-up was the date of the first GPP diagnosis. Primary analyses to assess demographics, clinical characteristics, treatments, flares and mortality were performed using the strict 3 criterion to increase the reliability of GPP diagnosis (Figure 1). The inclusion period for the primary analysis group was 1996–2019 to allow a minimum of a 2-year follow-up.

For comparison, two age- and sex-matched populations were formed: a PV population (ICD-10: L40.0) without GPP was identified from the Care Register for Health Care, and a population-based control group without GPP or PV was identified from the Digital and Population Data Services Agency. Each patient with GPP at the index date was matched by age and sex with 10 control participants with PV and 10 population-based control participants (1:10).

### Data sources

Primary and specialty healthcare diagnoses [ICD-10, International Classification of Primary Care (ICPC-2)] and healthcare resource utilization data were collected from The Register for Primary Care Visits and The Care Register for Health Care (The Finnish Institute for Health and Welfare), medication dispensations from The Register for Reimbursed Drugs (The Social Insurance Institution) and the dates of death from The Causes of Death Register (Statistics Finland).

### Study outcomes

#### Epidemiology

Period (1996–2021) and annual prevalences (2000–21) were calculated per 100 000 people by dividing the number of cases of GPP by an approximation of the Finnish population for that time and multiplying by 100 000. The overall incidence rate was calculated similarly for 2010–21 to ensure a minimum of a 10-year period with no prior history of GPP. Annual incidence rates were calculated between 2010 and 2021. Prevalence and incidence rates were analysed for all subgroups and presented with 95% confidence intervals (CIs).

#### Demographics, clinical characteristics and treatments

Demographics, comorbidities (Table S1; see Supporting Information) and use of GPP-related medications (Table S2; see Supporting Information) were compared between patients with GPP and matched control groups. As GPP had no medication reimbursement status in Finland during the study period, GPP-related medications were defined similar to as described in Löfvendahl *et al*.,^31^ assuming that psoriasis-related drugs were prescribed for GPP in the primary analysis group.

#### Generalized pustular psoriasis flares

GPP flares were defined as GPP recorded as a primary diagnosis during a hospitalization. Patients with GPP were stratified into groups with ≥1 flare(s) and without flares during the study period. Flare frequency was analysed both for prevalent and incident primary analysis groups (Figure 1).

#### Mortality

The mortality rate for GPP was compared with the PV and population-based control groups, and between patients with GPP with ≥1 flare vs. no flares during ­follow-up.

### Statistical analyses

Descriptive analyses included counts and proportions for categorical variables, and mean (SD) or median (interquartile range) for continuous variables. Conditional logistic regression was used for matched comparisons (GPP vs. control groups)^32^ and *t*-tests for unmatched comparisons (between case criteria). Mortality was analysed using the Kaplan–Meier method. GPP flare frequency was estimated with the Aalen–Johansen estimator, accounting for death as a competing risk. Associations between flares and potential triggers in the 5 years before the GPP index date (Tables S1, S2) were assessed using Cox regression, with time to flare as the outcome and censoring for death or end of follow-up. Flare–mortality associations were analysed via Kaplan–Meier and Cox models, adjusting for age and sex. Flares within 6 months were evaluated using the clone–censor–weight method to reduce immortal time bias.^33^

## Results

### Generalized pustular psoriasis population

Altogether, 655 patients with ≥1 recorded GPP diagnosis (base criterion) in specialty healthcare were identified during follow-up (Figure 1). The number of prevalent and incident cases decreased with stricter criteria (Figure 1). The number of eligible patients with GPP in the prevalent primary analysis group was 286, and the mean (SD) follow-up time was 11.1 (6.8) years.

### Prevalence and incidence

**Figure 2:**
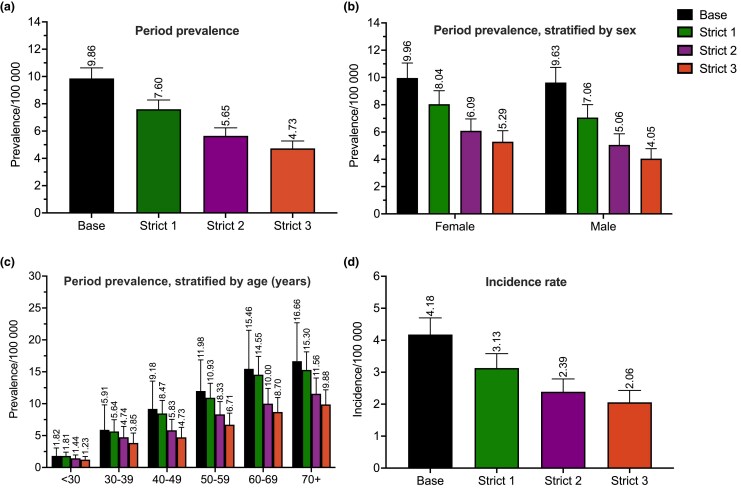
GPP period prevalence (1996–2021) and incidence (2010–2021) with 95% CIs with the different case criteria. (a) Prevalence for the entire study population, (b) prevalence stratified by sex, (c) prevalence stratified by age and (d) incidence rate. CI, confidence interval; GPP, generalized pustular psoriasis; base, ≥1 diagnosis in specialty care; strict 1, ≥1 diagnosis in dermatology clinic in specialty care; strict 2, ≥2 diagnoses in specialty care; strict 3, ≥2 diagnoses in dermatology clinic in specialty care.

Period prevalence was 9.9 per 100 000 individuals with the base criterion and 4.7 per 100 000 when applying the strict 3 criterion, during 1996–2021 (Figure 2a). With strict 3, the prevalence was 1.3-fold higher for women (women 5.3/100 000; men 4.1/100 000) (Figure 2b). Period prevalence was the highest in the 70+ years age group, irrespective of the criterion (Figure 2c). Additionally, the annual prevalence increased approximately 3.3-fold between 2000 and 2021, regardless of the criterion (Figure S1a; see Supporting Information).

The overall incidence of GPP during 2010–21 was 4.2 per 100 000 with base criterion and 2.1 per 100 000 with strict 3 criterion (Figure 2d). The mean annual incidence rate for GPP between 2010 and 2021 ranged from 0.48 to 0.51 per 100 000 with the base criterion, and from 0.30 to 0.36 per 100 000 with the strict 3 criterion (Figure S1b).

### Demographic and clinical characteristics

The proportion of women (59.1%, *n* = 169/286) was higher than in the GPP primary analysis group, and the mean (SD) age was 56.0 (17.9) years (Table 1). The most common comorbidities were psoriasis (73.4%, *n* = 210/286), hypertension (48.6%, *n* = 139/286), periodontal disease (30.4%, *n* = 87/286), type 2 diabetes (28.3%, *n* = 81/286) and upper respiratory tract infections (26.2%, *n* = 75/286). Most comorbidities evaluated in this study had a significantly higher prevalence in GPP compared with the population-based control group [Table 1; Table S3 (see Supporting Information)]. Eight comorbidities (of 37) were significantly more common in GPP compared with PV, the most common of which were psoriatic arthritis [GPP 23.8% (*n* = 68/286); PV 12.2% (*n* = 336/2752); *P* < 0.001], inflammatory polyarthropathies [GPP 23.4% (*n* = 67/286); PV 14.7% (404/2752); *P* < 0.001] and respiratory diseases [GPP 23.4% (*n* = 67/286); PV 15.4% (*n* = 424/2752); *P* < 0.01] (Table 1, Table S3).

**Table 1:** Demographic and clinical characteristics of the primary analysis group with generalized pustular psoriasis (strict 3, with and without flares) compared with population-based and psoriasis vulgaris control groups during follow-up

Variables	GPP			Population control (*n* = 2844)	PV control (*n* = 2752)
	Without flares (*n* = 163)	≥1 flare (*n* = 123)	All (*n* = 286)		
Sex					
Female	86 (52.8)	83 (67.5)^#^	169 (59.1)	1684 (59.2)	1608 (58.4)
Male	77 (47.2)	40 (32.5)^#^	117 (40.9)	1160 (40.8)	1144 (41.6)
Age at index date (years)					
Mean (SD)	53.9 (17.6)	58.7 (18.0)^#^	56.0 (17.9)	56.0 (17.9)	54.7 (17.0)
Median (IQR)	55.0 (42.0–67.0)	60.0 (48.0–73.0)^#^	57.0 (45.0–69.0)	57.0 (45.0–69.0)	56.0 (45.0–68.0)
Number of flares per patient per follow-up year					
Mean (SD)	0.0 (0.0)	0.74 (3.0)	0.32 (2.00)	NA	NA
Median (IQR)	0.0 (0.0–0.0)	0.18 (0.09–0.40)	0.0 (0.00–0.14)	NA	NA
Comorbidities^a^					
Psoriasis (PV/other/unspecified)^b^	116 (71.2)	94 (76.4)	210 (73.4)	12 (0.4)***	2752 (100)
Hypertension	81 (49.7)	58 (47.2)	139 (48.6)	1170 (41.1)*	1327 (48.2)
Periodontal disease	57 (35.0)	30 (24.4)	87 (30.4)	679 (23.9)*	772 (28.1)
Type 2 diabetes	45 (27.6)	36 (29.3)	81 (28.3)	497 (17.5)***	643 (23.4)
Other dermatitis	37 (22.7)	34 (27.6)	71 (24.8)	207 (7.3)***	730 (26.5)
Upper respiratory tract infections	55 (33.7)	20 (16.3)^###^	75 (26.2)	748 (26.3)	825 (30.0)
Psoriatic arthritis, unspecified	37 (22.7)	34 (25.2)	68 (23.8)	7 (0.2)***	336 (12.2)***
Dorsalgia	43 (26.4)	25 (20.3)	68 (23.8)	614 (21.6)	649 (23.6)
Respiratory diseases, unspecified	39 (23.9)	28 (22.8)	67 (23.4)	386 (13.6)***	424 (15.4)**
Inflammatory polyarthropathies, unspecified	38 (23.3)	29 (23.6)	67 (23.4)	171 (6.0)***	404 (14.7)***
Other soft tissue disorders	36 (22.1)	28 (22.8)	62 (22.4)	526 (18.5)	557 (20.2)
Neoplastic diseases, unspecified	29 (17.8)	35 (28.5)^#^	64 (22.4)	581 (20.4)	566 (20.6)
Arthralgia	36 (22.1)	27 (22.0)	63 (22.0)	538 (18.9)	665 (24.2)
Ischaemic heart diseases	28 (17.2)	34 (27.6)^#^	62 (21.7)	458 (16.1)*	434 (15.8)*
Atrial fibrillation	34 (20.9)	24 (19.5)	58 (20.3)	469 (16.5)	395 (14.4)*
Heart failure	28 (17.2)	29 (23.6)	57 (19.9)	291 (10.2)***	322 (11.7)***
Dyslipidaemias	40 (24.5)	17 (13.8)^#^	57 (19.9)	550 (19.3)	596 (21.7)
Psychiatric disorders, unspecified	31 (19.0)	23 (18.7)	54 (18.9)	472 (16.6)	515 (18.7)
Gastrointestinal disease, unspecified	20 (12.3)	11 (8.9)	31 (10.8)	265 (9.3)	328 (11.9)
Ophthalmological involvement, unspecified	19 (11.7)	10 (8.1)	29 (10.1)	208 (7.3)	247 (9.0)
GPP-related medications					
TCS	148 (90.8)	114 (92.7)	262 (91.6)	742 (26.1)***	2403 (87.3)*
Conventional systemic drugs	85 (52.1)	94 (76.4)^###^	179 (62.5)	42 (1.5)***	631 (22.9)***
SCS	78 (47.9)	80 (65.0)^##^	158 (55.2)	748 (26.3)***	1069 (38.8)***
Topical calcipotriols	76 (46.6)	77 (62.6)^##^	153 (53.5)	22 (0.8)***	1719 (62.5)**
Biologics	26 (16.0)	29 (23.6)	55 (19.2)	13 (0.5)***	139 (5.1)***
Emollients and antipsoriatics	27 (16.6)	26 (21.1)	53 (18.5)	69 (2.4)***	448 (16.3)
Apremilast	<5^c^	<5	8 (2.8)	0 (0)***	30 (1.1)*
Calcineurin inhibitors	<5	<5	<5	<5	13 (0.5)

Data are presented as *n* (%) unless otherwise stated. GPP, generalized pustular psoriasis; IQR, interquartile range; NA, not available; PV, psoriasis vulgaris; SCS, systemic corticosteroids; TCS, topical corticosteroids. ^a^Comorbidities with a prevalence rate of >10% are shown. Comorbidities with a prevalence rate ≤10% are provided in Table S3, and comorbidities with *n* < 5 or *n* = 0 for GPP are not tabulated (toxic liver disease with cholestasis, neutrophilic cholangitis, alcoholic fatty liver disease, cytomegaloviral infections, skin mycosis, Epstein–Barr viral infection). ^b^PV, ICD-10: L40.0; Psoriasis other, ICD-10: L40.8; psoriasis unspecified, ICD-10: L40.9. ^c^If the *n* number is <5 in one or both groups (‘without GPP flares’ and/or ‘≥1 GPP flare(s)’) both results are masked. *P < 0.05, **P < 0.01, ***P < 0.001 between all patients with GPP and the population-based/PV control group. ^#^*P* < 0.05, ^##^*P* < 0.01, ^###^*P* < 0.001 between the groups ‘without flares’ and ‘≥1 flares’.

### Treatments

The most common GPP-related medication group was topical corticosteroids [TCS; 91.6% (*n* = 262/286)], followed by conventional systemic drugs (62.5%, *n* = 179/286), systemic corticosteroids [SCS; 55.2% (*n* = 158/286)], topical calcipotriols (53.5%, *n* = 153/286) and biologics (19.2%, *n* = 55/286; see Table 1). The use of TCS (*P* < 0.05), conventional systemic drugs (*P* < 0.001), SCS (*P* < 0.001) and biologics (*P* < 0.001) was significantly more common in GPP compared with PV. The use of calcipotriols was more common in PV compared with GPP (*P* < 0.001). The use of individual medications is shown in Table S4 (see Supporting Information).

### Flares and associated risk factors

In total, 43.0% (*n* = 123/286) of patients with GPP experienced ≥1 flare(s) during follow-up. Compared with patients without flares, patients with flares were more often women [67.5% (*n* = 83/123) vs. 52.8% (*n* = 86/163); *P* < 0.05] and older [mean age 58.7 vs. 53.9 years; *P* < 0.05 (Table 1)]. The prevalence of most comorbidities was similar in both subgroups. Ischaemic heart disease [27.6% (*n* = 34/123) vs. 17.2% (*n* = 28/164); *P* < 0.05] and neoplastic diseases [28.5% (*n* = 35/123) vs. 17.8% (*n* = 29/163); *P* < 0.05] were more common in patients with flares vs. without flares, and upper respiratory tract infections [16.3% (*n* = 20/123) vs. 33.7% (*n* = 55/163); *P* < 0.001] and dyslipidaemias [13.8% (*n* = 17/123) vs. 24.5% (*n* = 40/163); *P* < 0.05] were less common. The use of conventional systemic drugs [76.4% (*n* = 94/123) vs. 52.1% (*n* = 85/163); *P* < 0.001], SCS [65.0% (*n* = 80/123) vs. 47.9% (*n* = 78/163); *P* < 0.01] and topical calcipotriols [62.6% (*n* = 77/123) vs. 46.6% (*n* = 76/163); *P* < 0.01] was significantly more common in patients with flares vs. without flares. The use of individual medications is shown in Table S3.

**Figure 3:**
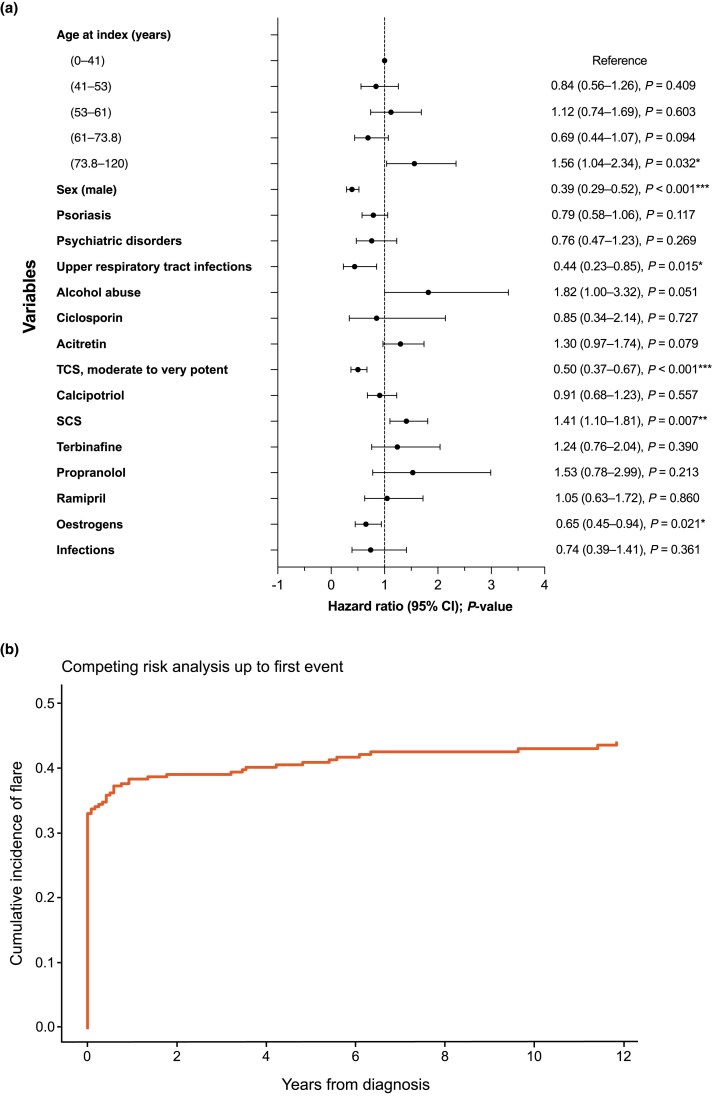
Risk factors and flare frequency in generalized pustular psoriasis (GPP). (a) Cox regression analysis of association between risk factors at baseline and GPP flares. (b) Proportion of patients with the first GPP flare estimated with the Aalen–Johansen estimator. CI, confidence interval; SCS, systemic corticosteroids; TCS, topical corticosteroids. *P < 0.05, **P < 0.01, ***P < 0.001.

Older age [>73.8 years: hazard ratio (HR) 1.56, 95% CI 1.04–2.34; *P* = 0.03] and use of SCS (HR 1.41, 95% CI 1.10–1.81; *P* = 0.007) at baseline were associated with a significantly higher risk of flares (Figure 3a). Conversely, having upper respiratory tract infections (HR 0.44, 95% CI 0.23–0.85; *P* = 0.015), use of TCS (HR 0.50, 95% CI 0.37–0.67; *P* < 0.001) and the use of oestrogens (HR 0.74, 95% CI 0.45–0.94; *P* = 0.021) were associated with a significantly lower risk of flares.

### Flare frequency

Altogether, 94 patients with GPP [32.9% of all patients (*n* = 286); 76.4% of patients with flares (*n* = 123)] had their first flare at the index date (Figure 3b, Table 2). Half of the patients (49.6%, *n* = 61/123) with flares experienced just one flare (Table 2). The cumulative analysis of the first flare event showed that most flares occurred within the first year of the first GPP diagnosis (38.1%), and the frequency plateaued by 10 years (42.7%) (Figure 3b). Flare frequency was essentially the same in the GPP incident population (*n* = 109), in which 29.4% (*n* = 32/109) of patients had their first flare at the index date and 36.7% (*n* = 40/109) had the first flare within 5 years.

**Table 2:** Number of generalized pustular psoriasis (GPP) flares and people experiencing flares per year of follow-up within the GPP prevalent primary analysis group (strict 3)

	Number of flares per follow-up year	Number of people^a^ with flare(s) per follow-up year
**Year from GPP index date**		
Index date = year 0	94	94
Year 1	72	46
Year 2	15	9
Year 3	12	8
Year 4	10	9
Year 5	8	6
Year 6	8	8
Year 7	11	6
Year 8	5	<5
Year 9	<5	<5
Year 10	6	<5
Year 11	<5	<5
Year ≥12	7	5
**Flare count**		**Number of people with flares during follow-up**
0		163
1		61
2		27
3		12
4		9
5		5
>5		9

Strict 3, ≥2 diagnoses in dermatology clinic in specialty care. ^a^Unique persons per one follow-up year.

### Mortality

**Figure 4:**
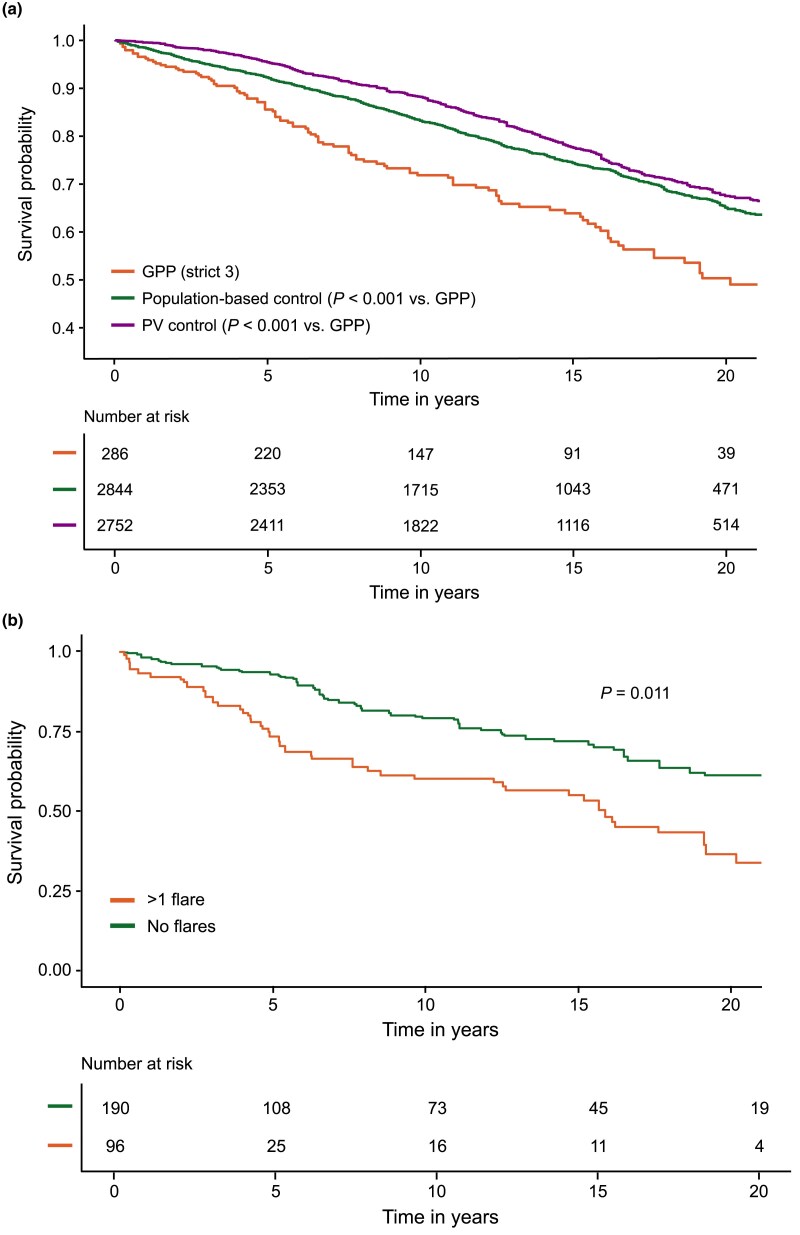
Kaplan–Meier estimates of survival rates for the (a) generalized pustular psoriasis (GPP) primary analysis group (strict 3, *n* = 286) and comparison with population-based (*n* = 2844) and psoriasis vulgaris (PV) (*n* = 2752) control groups, and (b) patients with GPP with (*n* = 123) and without (*n* = 163) flares (flares within 6 months of follow-up). Strict 3, ≥2 diagnoses in a dermatology clinic in specialty care.

The survival rate in the GPP group (86% at 5 years and 50% at 20 years) was significantly lower compared with the population-based control group (92% and 67%, respectively; *P* < 0.001) and the control group with PV (95% and 67%, respectively; *P* < 0.001) (Figure 4a). The survival rates did not differ between the case criteria (Figure S2; see Supporting Information). The mortality rate for GPP was 3.3% (95% CI 2.7–4.0) per 100 person-years. The most common main causes of death in the GPP group were ischaemic heart disease (7.7%, *n* = 22/286) and malignancies of the bronchus and lung (2.1%, *n* = 6/286) (Table S5; see Supporting Information). In addition to these, Alzheimer disease was a common cause of death in the population-based control group and the control group with PV [3.2% (*n* = 92/2844) and 2.4% (*n* = 67/2752), respectively].

The survival rate was significantly lower in patients with GPP with flares (73% at 5 years and 37% at 20 years) compared with those without flares (93% at 5 years and 61% at 20 years; *P* = 0.01) (Figure 4b). In the age- and sex-adjusted Cox model, having flares (within 6 months of index date) was associated with a 1.7-times higher risk of death (HR 1.7, 95% CI 1.08–2.60; *P* < 0.05).

## Discussion

This nationwide study using Finnish healthcare registers provides a comprehensive view of the epidemiology, demographics, clinical characteristics, treatments, flares and mortality of patients with GPP over 25 years. These results highlight the high disease burden, with comorbidities and medication more common in GPP than PV, and lower overall survival rate. Nearly half (43%) of patients with GPP experience flares, mostly occurring at occurring at the time first GPP diagnosis. Flares were associated with a 1.7-times higher risk of death.

Understanding GPP epidemiology has been challenging due its rarity, heterogeneous course and lack of validated diagnostic criteria.^34^ Expectedly, prevalence decreases with stricter criteria (e.g. from 9.1 to 3.2 per 100 000 in Sweden to 11.1 to 3.5 per 100 000 in Denmark).^14,16^ Similarly, this study found a decrease from 9.9 to 4.7 per 100 000 in Finland when using stricter criteria. Annual prevalence (1.1–3.9 per 100 000 with the strict 3 criterion) and incidence (0.09–0.36 per 100 000) rates aligned with previous studies from Sweden, Denmark, France, Spain and the UK.^9,11,14,16,35^ These findings underscore the importance of using consistent diagnostic criteria for GPP. Here, the strictest criteria were used for analyses to ensure diagnostic reliability.

The predominance of women (59.1%) and age distribution (mean 56.0 years and higher prevalence in the 60+ years age groups) found in the present study matched findings from Sweden, Denmark, the UK and Canada.^8,11,16,17,26,28^ In contrast, Japanese (61.5%) and French (54.4%) studies have reported a higher proportion of men and younger onset in Japan (45 years), possibly due to ethnic or diagnostic differences.^9,36^ In accordance with previous reports, patients with GPP had a generally higher prevalence of comorbidities, not only compared with the population-based control group, but also compared with the PV control group.^9,17,18,29^ Moreover, the use of psoriasis-specific medications, including biologics, was generally more frequent in GPP than in PV across this and previous studies, reflecting the greater the greater disease severity of GPP.^18,31,36^

The present study shows that, in Finland, the first GPP diagnosis often coincides with the first flare. These flares occurred mostly in the first year after initial diagnosis, which is in line with US and French studies showing that most first flares occur within a month of the index GPP diagnosis, and that almost all the flares (89.0–97.4%) occur within a year.^9,10^ These findings suggest that flares may begin with mild symptoms, emphasizing the importance of recognizing early signs of flares to allow timely diagnosis and treatment before the development of a potentially life-threatening flare episode.

In our cohort, 43.0% of patients with GPP experienced a flare, which is higher than reported in French (30.9%) and US (17.6%) studies.^14,28^ In a recent UK study, the proportion ranged from 39.3% to 62.6%, depending on flare definition.^11^ These differences may be explained by the longer ­follow-up time in our study (mean 11.1 years vs. 4.3–4.7 years) and variations in patient and flare identification criteria. Among patients with flares (*n* = 123), the mean flare rate was 0.74 per person-years, equalling approximately one flare every 1.5 years, which is in line with previous findings.^9,10,19^ In contrast, higher rates (1.4–2.9 flares per person) have been reported in the UK.^11^ A US study found a higher comorbidity burden in patients with flares, while in our cohort, only ischaemic heart disease and neoplastic conditions were more common, likely reflecting the older age of this subgroup.^10^ Similar to the US study, medication use was more frequent among patients with flares.^10^

Among the clinically relevant flare risk factors assessed in this study, SCS use showed the strongest association with flares (41% higher risk), which is consistent with previous studies.^8,19,21,22,37,38^ Corticosteroid use was notably higher in patients with flares (76.4%) vs. those without (52.1%), suggesting a need to consider alternative treatments. Although prior research has linked acitretin, terbinafine, ramipril and propranolol to GPP flares, these associations were not found in this study to be statistically significant, likely due to low usage rates.^19,22,39,40^ Interestingly, upper respiratory tract infections and use of TCS were associated with a lower risk of flares, although such findings have not been reported previously.^8,19,21^ These may reflect confounding factors: respiratory infections might be under-reported due to more severe concurrent symptoms, and TCS may be more common in milder cases, while SCS are used in more severe disease. Flares were also associated with an increased risk of death, likely due to life-threatening complications such as sepsis, acute respiratory distress or cardiovascular events.^15,41–43^ Reported GPP-related mortality in hospitalized patients ranges from 2% to 16%.^7,8,12,42,44^ The overall lower survival rate in patients with GPP vs. the matched control groups aligns with findings from Sweden and the UK.^11,23^ The observed mortality rate (3.3 deaths per 100 person-years) falls within previously reported ranges (from ≤0.5 to >2 deaths).^15^ In addition to flares, the higher comorbidity burden of patients with GPP likely contributes to reduced survival.

The main strengths of this study include its nationwide, population-based cohort, comparison with matched control groups, and comprehensive data enabling a detailed characterization of patients with GPP over a long follow-up period. Limitations include the potential inaccuracies in ICD-10 codes, as diagnosis may not be fully captured in the registers. To mitigate this, the strictest case definition was applied to identifying the primary GPP analysis group. Additionally, treatment analyses may not be GPP-specific, as GPP lacked a dedicated reimbursement status during the study period.

In conclusion, this study enhances understanding of the characteristics and high disease burden of GPP in Finland. The disease burden in terms of treatment use and mortality was most evident in patients with flares, who comprised almost half of the GPP population. These findings offer valuable insights into flare patterns and current clinical practice, supporting more effective GPP management and timely diagnosis and intervention.

## Supplementary Material

vzaf076_Supplementary_Data

## Data Availability

The data underlying this article cannot be shared publicly due to Finnish legislation protecting the privacy of the individuals, and restricting access to individual-level data only to persons named in the study permit. Aggregated study data will be shared on reasonable request to the corresponding author.
