# Supplementary material for: Epidemiology, clinical characteristics, flares and mortality of generalized pustular psoriasis: a nationwide register study in Finland
Source: Skin Health Dis. 2025 Oct 9;5(6):432–42. doi: 10.1093/skinhd/vzaf076 (PMC12648532; doi:10.1093/skinhd/vzaf076)
Supplement: vzaf076_Supplementary_Data [file vzaf076_supplementary_data.docx]

**Supporting Information**

**Epidemiology, clinical characteristics, flares and mortality of generalized pustular psoriasis – a nationwide register study in Finland**

Mirkka Koivusalo^1^, Xavier Teitsma^2^, Juha Mehtälä^1^, Aino Vesikansa^1^, Susanne Clemen Capion^3^, Laura Airaksinen^4^, Maria Grönman^4^, Rafael Pasternack^5^, Pauliina Nuutinen^6^, Laura Huilaja^7^

**Running title**: Epidemiology, clinical characteristics and flares of GPP in Finland

**Affiliations**

^1^MedEngine Oy, Helsinki, Finland

^2^Boehringer Ingelheim B.V., Amsterdam, the Netherlands

^3^Boehringer Ingelheim, Copenhagen, Denmark

^4^Boehringer Ingelheim Ky, Helsinki, Finland

^5^Tampere University Hospital and Faculty of Medicine and Health Technology, Tampere University, Tampere, Finland

^6^Helsinki University Hospital, Helsinki, Finland

^7^Research Unit of Clinical Medicine, University of Oulu and Medical Research Center, Oulu University Hospital, Oulu, Finland

***Corresponding author:**

Name: Mirkka Koivusalo

e-mail: [mirkka.koivusalo@medengine.fi](mailto:mirkka.koivusalo@medengine.fi)

**SUPPLEMENTARY TABLES**

**Table S1.** ICD-10 and ICPC-2 codes used for the selected comorbidities as recorded in primary and specialty healthcare.

| **Comorbidity** | **ICD-10 / ICPC-2 code** | **Description** |
| --- | --- | --- |
| Psoriasis^1^ | ICD-10: L40.0, L40.8, L40.9 | Psoriasis vulgaris, psoriasis other, psoriasis unspecified |
| Psoriatic arthritis, unspecified | ICD-10: L40.5, M07*, M09* | Psoriatic arthritis, psoriatic and enteropathic arthropathies, juvenile arthritis |
| Other dermatitis | ICD-10: L30 | Other dermatitis |
| Other soft tissue disorders | ICD-10: M79 | Other soft tissue disorders |
| Hypertension | ICD-10: I10-15; ICPC-2: K85-88 | Hypertensive diseases |
| Atrial fibrillation | ICD-10: I48; ICPC-2: K78 | Atrial fibrillation |
| Ischemic heart diseases | ICD-10: I20-25; ICPC-2: K74-76 | Ischemic heart diseases |
| Heart failure | ICD-10: I50; ICPC-2: K77 | Heart failure |
| Dyslipidaemias | ICD-10: I20-25; ICPC-2: T93 | Disorders of lipoprotein metabolism and other lipidaemias |
| Respiratory diseases, unspecified | ICD-10: J44, J45, J84.1, J96, J80-84; ICPC-2: R95, R96, R28, R88, R99 | COPD, asthma, pulmonary fibrosis, respiratory failure, other respiratory diseases affecting the interstitium |
| Allergic rhinoconjunctivitis | ICD-10: J30 | Allergic rhinoconjunctivitis |
| Allergic contact dermatitis | ICD-10: L23.9 | Allergic contact dermatitis |
| Obesity^1^ | ICD-10: E66; ICPC-2: T82-83 | Obesity |
| Type 1 diabetes | ICD-10: E10; ICPC-2: T89 | Type 1 diabetes mellitus |
| Type 2 diabetes | ICD-10: E11; ICPC-2: T90 | Type 2 diabetes mellitus |
| Dorsalgia | ICD-10: M54 | Dorsalgia |
| Thyroid associated diseases, unspecified | ICD-10: E03, E05, E21.0, E06; ICPC-2: T85-86 | Hypothyroidism, thyrotoxicosis (hyperthyroidism), primary hyperparathyroidism, thyroiditis |
| Gastrointestinal diseases, unspecified | ICD-10: K50, K51, K90.0, K57, K27, ICPC-2: D93-94 | Crohn’s disease, ulcerative colitis; celiac disease, diverticular disease of the intestine, peptic ulcer disease |
| Disease of the liver, unspecified | ICD-10: K71-76, ICPC-2: D97 | Toxic liver disease, toxic liver failure, hepatic failure, chronic hepatitis, cirrhosis and fibrosis of the liver, other inflammatory liver diseases, other disease of the liver including non-alcoholic fatty liver disease (NAFLD) |
| Disease of the liver^2^ | ICD-10: K70.0 | Alcoholic fatty liver disease^2^ |
| Disease of the liver^2^ | ICD-10: K71.0 | Toxic liver disease with cholestasis^2^ |
| Disease of the liver^2^ | ICD-10: K83.0 | Neutrophilic cholangitis^2^ |
| Periodontal disease, unspecified | ICD-10: K04.4, K04.5, K05.2-K05.6 ICPC-2: D19-20 | Acute apical periodontitis of pulpal origin, chronic apical periodontitis, acute periodontitis, chronic periodontitis, other periodontal disease, periodontal disease unspecified |
| Psychiatric disorders, unspecified^1^ | ICD-10: F31-34, F40-41, F43, F50, F20, F45.8, ICPC-2: P01-03, P27, P29, P72-77, P80 | Bipolar affective disorder, depressive episodes, recurrent depressive disorder, persistent mood disorders, phobic anxiety disorders, other anxiety disorders, reaction to severe stress, eating disorders, schizophrenia, suicidal ideation |
| Sleeping disorders | ICD-10: G47, ICPC-2: P06 | Sleeping disorders |
| Inflammatory polyarthropathies | ICD-10: M05-07, M13.0, ICPC2-: L88-91 | Seropositive rheumatoid arthritis, other rheumatoid arthritis, psoriatic and enteropathic arthropathies, other arthritis |
| Arthralgia | ICD-10: M25, ICPC-2: L20, L28-29, L70 | Arthralgia |
| Upper respiratory tract infections, unspecified^1^ | ICD-10: J00-06, ICPC-2: R74, R78-79 | Acute other respiratory infections |
| Infections: Chronic tonsillitis ^1^ | ICD-10: J35, ICPC-2: R76 | Chronic tonsillitis |
| Infections: Varicella-zoster virus^1^ | ICD-10: B01-02 | Varicella-zoster virus (chickenpox, shingles) |
| Infection: Epstein-Barr^1,2^ | ICD-10: B27.0 | Epstein-Barr virus (mononucleosis) |
| Infection: Streptococcal infection ^1^ | ICD-10: G00.2, J02-03 | Streptococcal infection: Streptococcal meningitis, pharyngitis, tonsillitis |
| Infection: Trichophyton rubrum ^1^ | ICD-10: B35 | Trichophyton rubrum: dermaphytosis |
| Infection: Cytomegalovirus^1,2^ | ICD-10: B27.1, B25 | Cytomegalovirus: cytomegaloviral mononucleosis, cytomegaloviral disease |
| Infection: Skin mycosis^1,2^ | ICD-10: B36.0 | Skin mycosis |
| Kidney diseases, unspecified | ICD-10: N17-N19, N00-N08; ICPC-2: U14, U70, U88 | Glomerular diseases, renal failure |
| Neoplastic diseases, unspecified | ICD-10: C*; ICPC-2: B72-75, D74-78, F74, H75, R84-86, L71, K72, N74-76, R84-86, S77-78, S81, T71-73, U75-79, X75-81, Y77-79 | Neoplastic diseases |
| Melanomas | ICD-10: C43-44; ICPC-2: S77 | Melanomas and other malignant neoplasms of the skin |
| Cerebrovascular diseases: cerebral infarction | ICD-10: I63 | Cerebral infarction |
| Ophthalmological involvement, unspecified | ICD-10: H10, H20, H30, ICPC-2: F70-73 | Conjunctivitis, iridocyclitis, chorioretinal inflammation |
| Oedema | ICD-10: R60 | Oedema |
| Osteoporosis | ICD-10: M80, M81; ICPC-2: L95 | Osteoporosis |
| Alcohol abuse^1^ | ICD-10: F10, ICPC-2: P15-16 | Alcohol-related disorders |

^1^Comorbidities considered as risk factors for GPP flare in Cox regression analysis

^2^Comorbidities with n<5 or n=0 for GPP.

COPD, chronic obstructive pulmonary disease; ICD-10, ICD-10, International Classification of Diseases, 10^th^ Revision; ICPC-2, International Classification of Primary Care, 2nd edition; NAFLD, non-alcoholic fatty liver disease.

**Table S2.** ATC codes used to define GPP-related and other medications

| **Type of Drug** | **ATC code** | **Active substance** |
| --- | --- | --- |
| **CONVENTIONAL SYSTEMIC DRUGS** | | |
| All psoriasis-related systemic conventionals | ATC codes below as a group | All selected conventionals, unspecified |
| Conventional systemic IS | L01BA01  L04AX03 | Methotrexate |
| Conventional systemic IS | L04AX01 | Azathioprine |
| Conventional systemic IS | L04AD01 | Cyclosporine^1^ |
| Conventional systemic IS | [L04AA06](http://en.wikipedia.org/wiki/ATC_code_L04) | Mycophenolate mofetil^2^ |
| Antipsoriatics for systemic use/Retinoids | D05BB02 | Acitretin^1^ |
| Antipsoriatics for systemic use/Psoralens | D05BA* | Psoralens for systemic use, unspecified |
| **APREMILAST** |  |  |
|  | L04AA32 | Apremilast |
| **TOPICAL CORTICOSTEROIDS (TCs)** | | |
| TCS^1^ | ATC codes below as a group | Topical CS, unspecified^1^ |
| TCS | D07AB* | Moderately potent topical CS |
| TCS | D07AC* | Potent topical CS |
| TCS | D07AD* | Very potent topical CS |
| **TOPICAL CALCIPOTRIOLS** | | |
| Antipsoriatics for topical use/Topical calcipotriol^1^ | ATC codes below as a group | Calcipotriols, unspecified^1^ |
| Antipsoriatics for topical use/Topical calcipotriol | D05AX02 | Calcipotriol |
| Antipsoriatics for topical use/Topical calcipotriol | D05AX52 | Calcipotriol, combinations |
| **EMOLLIENTS AND ANTIPSORIATICS** | | |
| Emollients and antipsoriatics | ATC codes below as a group | Emollients and antipsoriatics, unspecified |
| Emollients and protectives | D02AB* | [Zinc](http://en.wikipedia.org/wiki/Zinc) products^2^ |
| Emollients and protectives | D02AC* | Soft paraffin and fat products^2^ |
| Emollients and protectives | D02AE* | Carbamide products |
| Emollients and protectives | D02AF* | [Salicylic acid](http://en.wikipedia.org/wiki/Salicylic_acid) preparations^2^ |
| Emollients and protectives | D02AX* | Other emollients and protectives |
| Antipsoriatics for topical use | D05AA* | Tars^2^ |
| Antipsoriatics for topical use | D05AC* | Antracen derivatives, unspecified^2^ |
| Antipsoriatics for topical use | D05AD* | Psoralens for topical use, unspecified^2^ |
| **CALCINEURIN INHIBITORS** |  |  |
| Calcineurin inhibitors^2^ | ATC codes below as a group | Calcineurin inhibitors, unspecified |
| Calcineurin inhibitors^2^ | D11AH01 | Tacrolimus |
| Calcineurin inhibitors^2^ | D11AH02 | Pimecrolimus |
| **BIOLOGICS** |  |  |
| Biologics | ATC codes below as a group | Biologics, unspecified |
| T-cell deactivator | L04AA24 | Abatacept^2^ |
| CD11a antagonist | L04AA21 | Efalizumab^2^ |
| TNF-alpha inhibitor | L04AB01 | Etanercept |
| TNF-alpha inhibitor | L04AB02 | Infliximab^1,2^ |
| TNF-alpha inhibitor | L04AB04 | Adalimumab^1^ |
| TNF-alpha inhibitor | L04AB06 | Golimumab^1^ |
| IL-12/23 antagonist | L04AC05 | Ustekinumab^1^ |
| TNF-alpha inhibitor | L04AB05 | Certolizumab pegol |
| IL-1-R antagonist | L04AC03 | Anakinra^2^ |
| IL-6-R antagonist | L04AC07 | Tocilizumab^2^ |
| IL-17 antagonist | L04AC10 | Secukinumab |
| CD20 antagonist | L01XC02 | Rituximab |
| IL-23 antagonist | L04AC16 | Guselkumab |
| IL-23A antagonist | L04AC18 | Risankizumab^2^ |
| IL17A-R antagonist | L04AC12 | Brodalumab^2^ |
| IL-17A antagonist | L04AC13 | Ixekizumab |
| **SYSTEMIC CORTICOSTEROIDS (SCs)** | | |
| SCS^1^ | ATC codes below as a group | Systemic CS, unspecified^1^ |
| SCS | H02AB01 | Betamethasone^2^ |
| SCS | H02AB02 | Dexamethasone |
| SCS | H02AB04 | Methylprednisolone |
| SCS | H02AB06 | Prednisolone |
| SCS | H02AB07 | Prednisone |
| SCS | H02AB09 | Hydrocortisone |
| **OTHER MEDICATIONS: RISK FACTORS FOR GPP FLARES** | | |
| Antifungals | D01BA02 | Terbinafine^1^ |
| Psychoanaleptics | N06AX12 | Bupropion^1^ |
| Beta blockers | C07BA05, C07AA05 | Propranolol^1^ |
| Agents acting on the renin-angiotensin system | C09BA05, C09AA05 | Ramipril^1^ |
| Immunostimulants | L03AB* | Interferons^1,2^ |
| Sex hormones and modulators | G03C* | Oestrogens^1^ |

*^1^ Medications considered as risk factors for GPP flare in Cox regression analysis*

^2^ Medications with n<5 or n=0 for GPP.

ATC, Anatomical Therapeutic Chemical; CD, cluster of differentiation; GPP, generalized pustular psoriasis; IL, interleukin; IS, immunosuppressants; SCS, systemic corticosteroid; TCS, topical corticosteroid; TNF, tumour necrosis factor, strict 3, >2 diagnoses in a dermatology clinic in specialty care.

**Table S3**. Comorbidities with prevalence < 23% within the GPP primary analysis group (strict 3, with and without flares) and comparison with population-based and PV control groups during the follow-up.

| **Variables** | **GPP** | | | **Population control (N=2844)** | **PV control**  **(N=2752)** |
| --- | --- | --- | --- | --- | --- |
|  | **Without flares**  **(N=163)** | **>1 flare**  **(N=123)** | **All**  **(N=286)** |  |  |
| **Comorbidities^1^** | **n (%)** | **n (%)** | **n (%)** | **n (%)** | **n (%)** |
| Other soft tissue disorders | 36 (22.1%) | 28 (22.8%) | 62 (22.4%) | 526 (18.5%) | 557 (20.2%) |
| Neoplastic diseases, unspecified | 29 (17.8%) | 35 (28.5%)^#^ | 64 (22.4%) | 581 (20.4%) | 566 (20.6%) |
| Arthralgia | 36 (22.1%) | 27 (22.0%) | 63 (22.0%) | 538 (18.9%) | 665 (24.2%) |
| Ischemic heart diseases | 28 (17.2%) | 34 (27.6%)^#^ | 62 (21.7%) | 458 (16.1%)^*^ | 434 (15.8%)^*^ |
| Atrial fibrillation | 34 (20.9%) | 24 (19.5%) | 58 (20.3%) | 469 (16.5%) | 395 (14.4%)^*^ |
| Heart failure | 28 (17.2%) | 29 (23.6%) | 57 (19.9%) | 291 (10.2%)^***^ | 322 (11.7%)^***^ |
| Dyslipidaemias | 40 (24.5%) | 17 (13.8%)^#^ | 57 (19.9%) | 550 (19.3%) | 596 (21.7%) |
| Psychiatric disorders, unspecified | 31 (19.0%) | 23 (18.7%) | 54 (18.9%) | 472 (16.6%) | 515 (18.7%) |
| Gastrointestinal disease, unspecified | 20 (12.3%) | 11 (8.9%) | 31 (10.8%) | 265 (9.3%) | 328 (11.9%) |
| Ophthalmological involvement, unspecified | 19 (11.7%) | 10 (8.1%) | 29 (10.1%) | 208 (7.3%) | 247 (9.0%) |
| Thyroid diseases, unspecified | 16 (9.8%) | 11 (8.9%) | 27 (9.4%) | 243 (8.5%) | 266 (9.7%) |
| Kidney diseases, unspecified | 11 (6.7%) | 16 (13.0%) | 27 (9.4%) | 161 (5.7%)^*^ | 202 (7.3%) |
| Osteoporosis | 14 (8.6%) | 12 (9.8%) | 26 (9.1%) | 135 (4.7%)^**^ | 107 (3.9%)^***^ |
| Alcohol abuse | 15 (9.2%) | 10 (8.1%) | 25 (8.7%) | 116 (4.1%)^***^ | 230 (8.4%) |
| Sleeping disorders, unspecified | 16 (9.8%) | 6 (4.9%) | 22 (7.7%) | 211 (7.4%) | 274 (10.0%) |
| Oedema | 15 (9.2%) | 7 (5.7%) | 22 (7.7%) | 146 (5.1%) | 159 (5.8%) |
| Obesity | 11 (6.7%) | 10 (8.1%) | 21 (7.3%) | 116 (4.1%) | 198 (7.2%) |
| Cerebral infarction | 10 (6.1%) | 11 (8.9%) | 21 (7.3%) | 164 (5.8%) | 195 (7.1%) |
| Liver diseases, unspecified | 6 (3.7%) | 11 (8.9%) | 17 (5.9%) | 41 (1.4%)^***^ | 101 (3.7%) |
| Type 1 diabetes | 10 (6.1%) | 5 (4.1%) | 15 (5.2%) | 38 (1.3%)^***^ | 51 (1.9%)^***^ |
| Melanomas | 8 (4.9%) | 7 (5.7%) | 15 (5.2%) | 154 (5.4%) | 215 (7.8%) |
| Streptococcal infections | 7 (4.3%) | 5 (4.1%) | 12 (4.2%) | 76 (2.7%) | 92 (3.3%) |
| Allergic contact dermatitis | <5^2^ | <5 | 10 (3.5%) | 19 (0.7%)^***^ | 85 (3.1%) |
| Varicella zoster | <5 | <5 | 10 (3.5%) | 72 (2.5%) | 97 (3.5%) |
| Trichophyton rubrum | <5 | <5 | 9 (3.1%) | 82 (2.9%) | 167 (6.1%) |
| Allergic rhinoconjunctivitis | <5 | <5 | 7 (2.4%) | 72 (2.5%) | 103 (3.7%) |
| Chronic tonsilitis | <5 | <5 | 5 (1.7%) | 26 (0.9%) | 24 (0.9%) |

* p<0.05, **p< 0.01, *** p<0.001 between all GPP patients and the population-based/PV control group

^#^ p<0.05, ^##^ p<0.01, ^###^ p<0.001 between the groups ‘without flares’ and ‘>1 flares’

^1^Comorbidities with n<5 or n=0 for GPP are not tabulated (toxic liver disease with cholestasis, neutrophilic cholangitis, alcoholic fatty live disease, cytomegaloviral infections, skin mycosis, Epstein-Barr viral infection).

GPP, generalized pustular psoriasis; PV, psoriasis vulgaris; strict 3, >2 diagnoses in a dermatology clinic in specialty care.

**Table S4.** Use of individual GPP-related medications and medications as potential risk factors for GPP flares within the GPP primary analysis group (strict 3, with and without flares) and comparison with population-based and PV control groups during the follow-up.

| **Medication^1^** | **GPP** | | | **Population control (N=2844)** | **PV control**  **(N=2752)** |
| --- | --- | --- | --- | --- | --- |
|  | **Without flares**  **(N=163)** | **>1 flare**  **(N=123)** | **All**  **(N=286)** |  |  |
| **TCSs, unspecified^3^** | 148 (90.8%) | 114 (92.7%) | 262 (91.6%) | 742 (26.1%)^***^ | 2403 (87.3%)^*^ |
| TCSs, moderate | 65 (39.9%) | 79 (64.2%)^###^ | 144 (50.3%) | 323 (11.4%)^***^ | 1188 (43.2%)^*^ |
| TCSs, potent | 125 (76.7%) | 105 (85.4%) | 230 (80.4%) | 521 (18.3%)^***^ | 2040 (74.1%)^*^ |
| TCSs, very potent | 77 (47.2%) | 51 (41.5%) | 128 (44.8%) | 145 (5.1%)^***^ | 1043 (37.9%)^*^ |
| **Conventional systemic drugs** | 85 (52.1%) | 95 (77.2%)^###^ | 179 (62.5%) | 42 (1.5%)^***^ | 631 (22.9%)^***^ |
| Acitretin^3^ | 60 (36.8%) | 71 (57.7%)^###^ | 131 (45.8%) | 7 (0.2%)^***^ | 524 (19.0%)^***^ |
| Methotrexate | 61 (37.4%) | 64 (52.0%)^#^ | 125 (43.7%) | 55 (1.9%)^***^ | 621 (22.6%)^***^ |
| Cyclosporine^3^ | 19 (11.7%) | 24 (19.5%) | 43 (15.0%) | <5 | 35 (1.3%)^***^ |
| Azathioprine | <5^2^ | <5^2^ | 6 (2.1%) | 12 (0.4%)^**^ | 33 (1.2%) |
| **Apremilast** | <5 | <5 | 8 (2.8%) | 0 (0%)^***^ | 30 (1.1%)^*^ |
| **SCSs, unspecified^3^** | 78 (47.9%) | 80 (65.0%)^##^ | 158 (55.2%) | 748 (26.3%)^***^ | 1069 (38.8%)^***^ |
| Prednisolone | 70 (42.9%) | 72 (58.5%)^##^ | 142 (49.7%) | 621 (21.8%)^***^ | 906 (32.9%)^***^ |
| Prednisone | 12 (7.4%) | 12 (9.8%) | 24 (8.4%) | 96 (3.4%)^***^ | 159 (5.8%) |
| Methylprednisolone | 7 (4.3%) | 6 (4.9%) | 13 (4.5%) | 96 (3.4%) | 120 (4.4%) |
| Dexamethasone | <5 | <5 | 9 (3.1%) | 76 (2.7%) | 101 (3.7%) |
| Hydrocortisone | <5 | <5 | 6 (2.1%) | 12 (0.4%)^***^ | 11 (0.4%)^***^ |
| **Topical calcipotriols, unspecified^3^** | 76 (46.6%) | 77 (62.6%)^##^ | 153 (53.5%) | 22 (0.8%)^***^ | 1719 (62.5%)^**^ |
| Calcipotriol, combinations | 56 (34.4%) | 59 (48.0%)^#^ | 115 (40.2%) | 19 (0.7%)^***^ | 1510 (54.9%)^***^ |
| Calcipotriol | 42 (25.8%) | 45 (36.6%)^#^ | 87 (30.4%) | 7 (0.2%)^***^ | 659 (23.9%)^*^ |
| **Biologics, unspecified** | 26 (16.0%) | 29 (23.6%) | 55 (19.2%) | 13 (0.5%)^***^ | 139 (5.1%)^***^ |
| Adalimumab^3^ | 12 (7.4%) | 15 (12.2%) | 27 (9.4%) | 6 (0.2%)^***^ | 57 (2.1%)^***^ |
| Etanercept | 7 (4.3%) | 10 (8.1%) | 17 (5.9%) | 8 (0.3%)^***^ | 19 (0.7%)^***^ |
| Ustekinumab^3^ | 8 (4.9%) | 8 (6.5%) | 16 (5.6%) | 0 (0%)^***^ | 37 (1.3%)^**^ |
| Secukinumab | 6 (3.7%) | 8 (6.5%) | 14 (4.9%) | 0 (0%)^***^ | 33 (1.2%)^***^ |
| Guselkumab | <5 | <5 | 9 (3.1%) | 0 (0%)^***^ | 19 (0.7%)^***^ |
| Golimumab^3^ | <5 | <5 | 5 (1.7%) | 6 (0.2%)^***^ | 7 (0.3%)^***^ |
| Ixekizumab | <5 | <5 | 5 (1.7%) | 0 (0%)^***^ | 17 (6%)^*^ |
| **Emollients and antipsoriatics, unspecified** | 27 (16.6%) | 26 (21.1%) | 53 (18.5%) | 69 (2.4%)^***^ | 448 (16.3%) |
| Carbamide | 19 (11.7%) | 17 (13.8%) | 36 (12.6%) | 50 (1.8%)^***^ | 323 (11.7%) |
| Other emollients | 12 (7.4%) | 10 (8.1%) | 22 (7.7%) | 20 (0.7%)^***^ | 140 (5.1%) |
| **Calcineurin inhibitors, unspecified** | <5 | <5 | <5 | <5 | 13 (0.5%) |
| Pimecrolimus | <5 | <5 | <5 | <5 | 11 (0.4%) |
| Tacrolimus | <5 | <5 | 0 (0%) | 0 (0%) | 6 (0.2%) |
| **Potential risk factors for GPP flares** |  |  |  |  |  |
| Agents acting on the renin-angiotensin system: ramipril^3^ | 19 (11.7%) | 25 (20.3%)^#^ | 44 (15.4%) | 385 (13.5%) | 404 (14.7%) |
| Beta-blockers: propranolol^3^ | 15 (9.2%) | 8 (6.5%) | 23 (8.0%) | 194 (6.8%) | 196 (7.1%) |
| Antifungals: terbinafine^3^ | 16 (9.8%) | 7 (5.7%) | 23 (8.0%) | 186 (6.5%) | 324 (11.8%) |
| Psychoanaleptics: bupropion^3^ | <5 | <5 | 6 (2.1%) | 35 (1.2%) | 35 (1.3%) |
| Oestrogens^3^ | 22 (13.5%) | 21 (17.1%) | 43 (15.0%) | 576 (20.3%)^*^ | 485 (17.6%) |

* p<0.05, **p< 0.01, *** p<0.001 between GPP and the population-based/PV control group

^#^ p<0.05, ^##^ p<0.01, ^###^ p<0.001 between the groups ‘no GPP flares’ and ‘>1 GPP flare(s)’

^1^Medications with n<5 or n=0 for GPP are not tabulated: betamethasone, mycophenolate mofetil, certolizumab, abatacept, efalizumab, tocilizumab, risankizumab, brodalumab, anakinra, infliximab, tars, antracenes, interferons, topical psoralens.

^2^If the n number is <5 in one or both of the groups (‘no GPP flares’ and/or ‘>1 GPP flare(s)’) both results are masked.

^3^Medications considered as risk factors for GPP flare in Cox regression analysis

GPP, generalized pustular psoriasis; PV, psoriasis vulgaris; SCS, systemic corticosteroid; TCS, topical corticosteroid; strict 3, >2 diagnoses in dermatology clinic in specialty care; strict 3

**Table S5.** The number of GPP flares and persons with flares per year of follow-up within the GPP prevalent primary analysis group (strict 3).

|  | **Number of flares per follow-up year** | **Number of persons^1^ with flare(s) per follow-up year** |
| --- | --- | --- |
| **Year from GPP index date** |  |  |
| Index date = year 0 | 94 | 94 |
| Year 1 | 72 | 46 |
| Year 2 | 15 | 9 |
| Year 3 | 12 | 8 |
| Year 4 | 10 | 9 |
| Year 5 | 8 | 6 |
| Year 6 | 8 | 8 |
| Year 7 | 11 | 6 |
| Year 8 | 5 | <5 |
| Year 9 | <5 | <5 |
| Year 10 | 6 | <5 |
| Year 11 | <5 | <5 |
| Year >12 | 7 | 5 |
| **Flare count** |  | **Number of persons with flares during follow-up** |
| 0 |  | 163 |
| 1 |  | 61 |
| 2 |  | 27 |
| 3 |  | 12 |
| 4 |  | 9 |
| 5 |  | 5 |
| >5 |  | 9 |

^1^Unique persons per one follow-up year.

GPP, generalized pustular psoriasis; strict 3, >2 diagnoses in dermatology clinic in specialty care.

**Table S6.** The most common main causes of death for GPP primary analysis group (strict 3) and comparison with population-based and PV control groups during the follow-up.

|  | **GPP**  **(N=286)** | **Population control (N=2844)** | **PV control**  **(N=2752)** |
| --- | --- | --- | --- |
| **Most common causes of death** |  |  |  |
| Alzheimer’s disease (ICD-10: G30*) | N/A | 92 (3.2%) | 67 (2.4%) |
| Chronic ischemic heart disease (ICD-10: I25*) | 22 (7.7%) | 71 (2.5%) | 72 (2.7%) |
| Malignant neoplasm of bronchus and lung (ICD-10: C34*) | 6 (2.1%) | 26 (0.9%) | 29 (1.1%) |
| Acute myocardial infarction (ICD-10: I21*) | 5 (1.8%) | 56 (2.0%) | N/A |
| Psoriasis (ICD-10: L40*) | 5 (1.8%) | N/A | N/A |
| Hypertensive heart disease (ICD-10: I11*) | N/A | 25 (0.9%) | N/A |
| Cerebral infarction (ICD-10: I63*) | N/A | N/A | 27 (1.0%) |

ICD-10, international classification of diseases, 10th edition; GPP, generalized pustular psoriasis; PV, psoriasis vulgaris; strict 3, >2 diagnoses in dermatology clinic in specialty care.

**SUPPLEMENTARY FIGURES**

**Supplementary Figure 1.** GPP annual prevalence and incidence rates with 95% CIs for the different diagnostic criteria. A) Annual prevalence rate between 2000 and 2021. B) Annual incidence rate between 2010 and 2021.

CI, confidence interval; GPP, generalized pustular psoriasis; base, >1 diagnosis in specialty care; strict 1, >1 diagnosis in dermatology clinic in specialty care, strict 2, >2 diagnoses in specialty care; strict 3, >2 diagnoses in dermatology in specialty care.

**Supplementary Figure 2.** Kaplan-Meier estimates of survival rates for GPP subgroups of different case criteria.

GPP, generalized pustular psoriasis; base, >1 diagnosis in specialty care; strict 1, >1 diagnosis in dermatology clinic in specialty care, strict 2, >2 diagnoses in specialty care; strict 3, >2 diagnoses in dermatology in specialty care.
